# Exploring adapted water polo: a feasibility study on improving psychosocial recovery in women post-breast cancer treatment

**DOI:** 10.3389/fspor.2026.1910555

**Published:** 2026-09-30

**Authors:** S. Cuvelier, C. Goetgheluck-Villaron, C. Zemmour, S. Bernasconi, A. Tallet, A. Arthur, P. Dantin, P. Viens, M. Cohen, S. Calvin

**Affiliations:** 1 Management Sport Cancer Laboratory UR20122035V, Aix Marseille University and Paoli Calmettes Institute, Marseille, France; 2 Department of Clinical Research and Innovations, Paoli Calmettes Institute, Marseille, France; 3 Department of Radiotherapy, Paoli Calmettes Institute, Marseille, France; 4 Department of Surgery, Paoli Calmettes Institute, Marseille, France

**Keywords:** cancer, fatigue, quality of life, team-based sports, water exercise

## Abstract

**Purpose:**

This study aimed to evaluate the feasibility and acceptability of an adapted water polo programme (Aqua polo) for women following breast cancer treatment. Exploratory secondary objectives were to examine changes in cancer-related fatigue (CRF), quality of life, post-traumatic growth, physical function and participants’ psychosocial experiences. The intervention was conducted in a municipal swimming pool within a renowned local club, as part of a community-based research collaboration between a cancer center and a sport psychology laboratory.

**Methods:**

A 16-week intervention was implemented, with participants engaging in structured weekly sessions to evaluate both physical and psychosocial outcomes. Fifteen participants took part in weekly 2-hour Aqua polo sessions, supervised by a multidisciplinary team. A mixed-methods approach was used to assess feasibility (session adherence), physical improvements (aerobic capacity, muscle mass), and psychosocial dimensions, including CRF (R-PFS), quality of life (QLQ BR23), post-traumatic growth (PTG-I), and relational dynamics within the sporting environment (through semi-structured interviews). Quantitative data were analyzed using descriptive statistics and mixed linear models; qualitative interviews were coded inductively using grounded theory principles and NVivo software.

**Results:**

The programme achieved a high adherence rate, with 87% of participants attending at least 60% of the sessions. Exploratory analyses showed increases in aerobic capacity (*p* = 0.009), muscle mass (*p* = 0.01) and quality of life (*p* = 0.04), whereas no significant changes were observed in cancer-related fatigue. Participants described positive psychosocial experiences, including greater self-confidence, renewed competence and improved body image. Qualitative findings highlighted the importance of social interactions, team cohesion and the coach–athlete relationship in fostering engagement and perceived psychosocial recovery.

**Conclusion:**

This feasibility study demonstrates that an adapted water polo programme is feasible and well accepted by women following breast cancer treatment. Exploratory findings suggest potential benefits for physical function and psychosocial well-being, while supporting the development of future adequately powered controlled studies evaluating team-based aquatic interventions in cancer rehabilitation.

**Clinical Trial Registration:**

https://clinicaltrials.gov/study/NCT04235946, identifier: NCT04235946. French national registry identifier: ID-RCB 2019-A03003-54.

## Introduction

According to Global Cancer Observatory (GCO) projections, the global incidence of breast cancer in women is expected to increase by one million cases by 2040 (1). With rising remission rates (2), attention is increasingly turning to survivors’ quality of life, which is often compromised by the chronic nature of cancer-related fatigue (CRF) (3, 4). CRF is a common, long-lasting, and disabling consequence of cancer and its treatment, persisting years after remission and significantly impairing quality of life due to its multidimensional impact on physical, cognitive, and emotional functioning (5). Affecting 56%–95% of patients post-treatment and persisting in 20%, CRF manifests as exhaustion, weakness, and lack of energy (6, 7), and is further compounded by limited social support in coping with long-term effects and recurrence-related fears (8). In this context, physical activity (PA), before and/or after breast cancer diagnosis, improves survival, reduces recurrence, and enhances physical capacity, supporting autonomy and QoL (9). Yet, PA levels often drop post-treatment, emphasizing the need for tailored programs. Aquatic activities are particularly beneficial for those post-mastectomies or undergoing hormone therapy, as they reduce pain and improve physical capacity (10, 11).

Beyond physiological gains, group cohesion and therapeutic alliance have been identified as key facilitators of sustained engagement in physical activity programs (12). Team-based sports, such as Dragon Boat racing, have shown positive effects on adherence and foster post-traumatic growth (PTG), a central component of psychosocial recovery (13). In this context, adapted water polo (Aqua polo) emerges as a relevant and innovative option. Although it has not yet been explored for therapeutic use, it is the only aquatic team sport that simultaneously offers the physical benefits of water-based movement and the psychosocial advantages of collective dynamics and coaching support.

This feasibility study primarily seeks to evaluate the demand for an Aqua Polo intervention among women post-breast cancer, based on participation and adherence indicators (14). Secondarily, it explores trends in physical, emotional, and psychological outcomes (including QoL, CRF, and PTG), and qualitatively investigates how perceived psychosocial support may influence the recovery experience.

## Materials & methods

The methodology of this study was extensively detailed in a protocol publication (15). Only the main features of the intervention will be reviewed here. This study was registered in the French national registry (ID-RCB: 2019-A03003-54) and in ClinicalTrials.gov (NCT04235946, registered January 17, 2020).

This study follows the framework proposed by Bowen et al. (14) and is designed as a feasibility study. The primary focus is on the feasibility domain of *demand*, assessed through participation and retention rates. *Acceptability* and *implementation* are explored qualitatively via semi-structured interviews, focusing on participants’ experience and perceived conditions of delivery. Finally, potential trends in QoL, CRF, physical capacity, and PTG are examined in an exploratory, non-confirmatory manner.

### Procedures

#### Study design

This prospective, interventional, monocentric, and non-randomized study aimed to evaluate the feasibility of an adapted water polo program, called “Aqua polo,” for women after breast cancer treatment. The intervention spanned a 16-week period, consisting of weekly 2-hour training sessions with an effective practice time of 90 min. Although the published protocol described a 20-week programme, its duration was reduced to 16 weeks because the intervention was postponed to maximise recruitment within the planned inclusion window, leaving insufficient time to complete the programme before the summer break and reduced coach availability. This modification did not affect the feasibility objectives or the overall study design. The program was delivered in a community-based setting, specifically in a municipal swimming pool, in collaboration with a well-established local water polo club.

#### Recruitment and screening

Participants were eligible for inclusion between 3 weeks and 3 months after completion of chemotherapy and/or radiotherapy. Ongoing hormone therapy and/or immunotherapy did not preclude participation. Participants who underwent surgery were additionally required to be at least 6 weeks post-operative. They were informed of the protocol during a face-to-face consultation with their oncology team, received a detailed information brochure, and, after providing written informed consent, were referred to the sports medicine centre for pre-inclusion physical assessments. Figure 1 illustrates participant flow from enrollment to analysis, all participants being retained in the intention-to-treat analysis.

**Figure 1:**
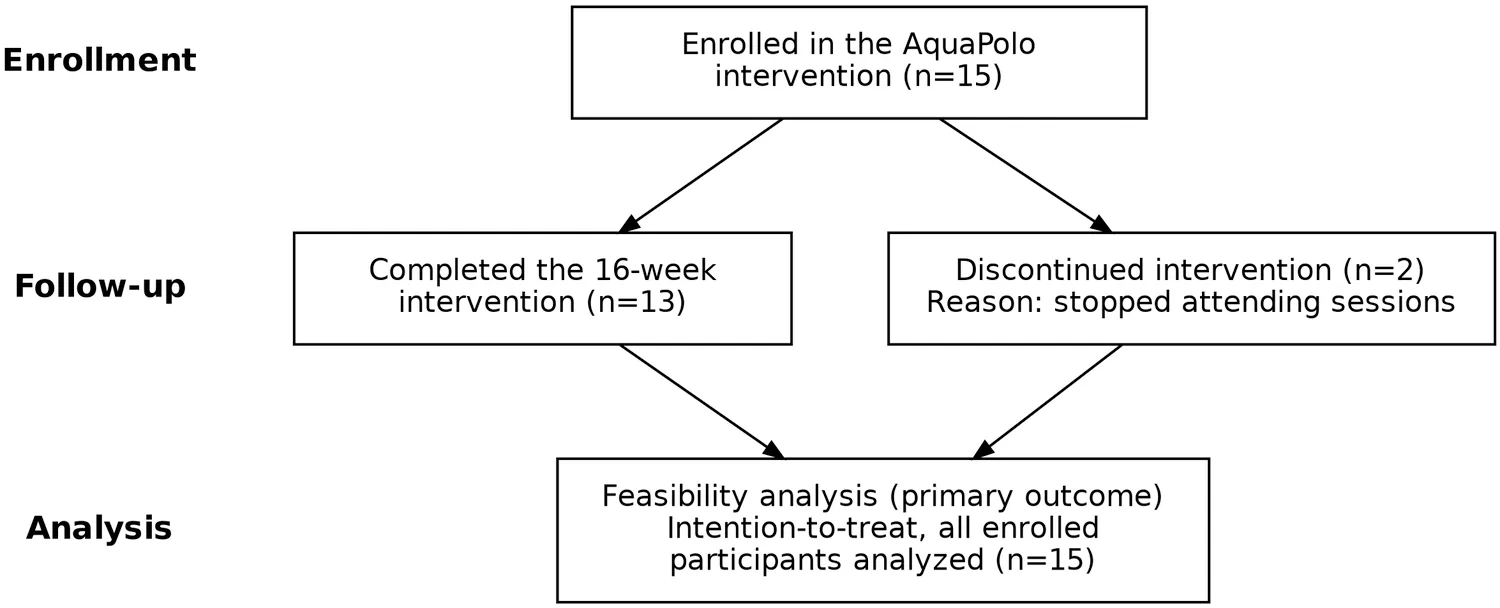
Participant flow diagram.

#### Coaching team

The participants were coached by a multidisciplinary team:

- Two high-level water polo coaches
- An adapted PA instructor
- A physiotherapist specializing in post-breast cancer care
- A technical manager from the water polo club
- A team of social psychology researchers

This group of professionals constituted the Aqua polo team staff.

#### Intervention process

The intervention's detailed progression, including physical and technical adjustments, is outlined in the methodological article (15). Training sessions gradually increased from 45 min to 1 h 30 during the final tournament, with technical and physical difficulty adjusted based on participant feedback. Regular staff meetings refined the program, emphasizing team cohesion through shared identity-building (e.g., sports equipment) and post-training snacks.

#### Adaptations and safety

Participants commencing the programme shortly after radiotherapy had completed skin healing and received medical clearance. Adverse events were prospectively collected from informed consent until 30 days after the final Aqua Polo session and graded according to severity, in accordance with the study safety protocol. No adverse or skin related serious events related to the intervention were reported.

Several adaptations were implemented to ensure the practice was accessible and safe:

- Use of smaller balls than those used by adult water polo teams.
- Reducing the area to limit movement in water.
- Modification of game rules, such as prohibiting touching an opponent holding the ball.
- Reduction of game time during matches, particularly during the final tournament.
- Systematic provision of flotation aids (aquagym buoys), at the discretion of the participants.

### Evaluations

The objectives and their evaluation criteria are summarized in Table 1.

**Table 1:** Correspondence between objectives and evaluation criteria.

Principal objective	Evaluation criteria
Feasibility	Percentage of patients completing the programme (> 60% of sessions completed).

Secondary objectives		Evaluation criteria
Physical measurements	Evaluate evolution of body composition	Fat/lean mass ratio obtain with impedance measurement (Balance Withings^ⓡ^) Body weight (Balance Withings^ⓡ^)
	Evaluate evolution of the amplitude of arm movement on the side of the treated breast	External rotation (elbow close to the body) and abduction of the shoulder in degrees
	Evaluate evolution of the aerobic capacity	3 min step test (16)
	Evaluate evolution of the strength of upper member and lower member	Quadriceps and grips strength evaluated by dynamometers
Psychosocial measurements	Evaluate evolution of post-traumatic growth	Questionnaire PTGI (17)
	Evaluate evolution of QoL	Questionnaire QLQ-BR23 (18)
	Evaluate evolution of fatigue	Questionnaire R-PFS (19)
	Evaluate the relation between coach and athlete	Questionnaire Cart-Q (20)
Interview	Evaluate psychological variables and the quality of the participant–coach relationship, with particular attention to the emotional, cognitive, and behavioural responses, as well as to assess participant satisfaction and key aspects of implementation	Semi-directive interview using a pre-established grid

PTGI, Posttraumatic Growth Inventory; QLQ-BR23, European Organisation for Research and Treatment of Cancer Quality of Life Questionnaire – Breast Cancer Module (EORTC QLQ-BR23); R-PFS, Revised Piper Fatigue Scale; CART-Q, Coach–Athlete Relationship Questionnaire.

The frequency of the measurements is summarized in Table 2.

**Table 2:** Measurements flow chart.

Assessment	Inclusion	W4	W8	W10	W12	W16/end of program
Inclusion						
Criteria for inclusion and non-inclusion	X					
Signature of informed consent	X					
Pregnancy test (if applicable)	X					
Visit to sports doctor to validate aptitude for aqua-polo	X					
Physical measurments						
Height	X					
Weight	X	X	X		X	X
Fat/lean mass	X	X	X		X	X
Amplitude of arm on side of treated breast	X			X		X
Step test 3 min	X			X		X
Handgrip strength	X			X		X
Quadriceps strength (dynamometer)	X			X		X
Scales and questionnaires						
QLQ-BR23	X			X		X
R-PFS	X	X	X		X	X
CART-Q				X		X
PTGI	X					X
Interviews						
Semi-directive interviews	X			X		X

QLQ-BR23, European Organisation for Research and Treatment of Cancer Quality of Life Questionnaire – Breast Cancer Module (EORTC QLQ-BR23); R-PFS, Revised Piper Fatigue Scale; CART-Q, Coach–Athlete Relationship Questionnaire; PTGI, Posttraumatic Growth Inventory.

### Data analysis

This study employs a mixed-methods design to assess the feasibility of the intervention, primarily focusing on demand, and to explore potential outcomes and underlying processes (21). Integrating qualitative and quantitative data enables comprehensive analysis and enhances interpretation of complex realities (22, 23).

Statistical analyses, performed using SAS® 9.4 software at the significance level *α*=0.05, included descriptive statistics for qualitative variables and means (±SD), medians [min-max] for quantitative endpoints.

Feasibility was assessed by the percentage of patients completing ≥60% of sessions, with a target 90%-power to reject at the significance level 5% the null hypothesis of a 40% unacceptable rate, assuming a 70% expected rate. Secondary endpoints were analyzed using linear mixed-effects models including time as a fixed effect and participant as a random effect. An unstructured covariance matrix was specified and parameters were estimated using restricted maximum likelihood (REML). Because all secondary analyses were predefined as exploratory, no adjustment for multiple comparisons was applied.

The qualitative analysis adopted an inductive approach informed by constructivist grounded theory principles (24) in which categories are co-constructed through an iterative, interpretative process. An initial theoretical framework informed the design of a broad, non-directive interview guide, but the analysis remained inductive, allowing themes to emerge from the data without imposing predefined constructs. A total of 45 semi-structured interviews were conducted with the 15 participants at three time points during the programme. Interviews were transcribed and analyzed with NVivo software used as a support tool to assist with data organization and coding. The first author coded the full dataset, with initial codes applied to text segments (25) and subsequently refined into categories through peer debriefing with two additional social psychology researchers. Reflexive memos were maintained throughout the analysis to document analytic decisions and researchers’ positioning, and categories were iteratively refined to identify overarching patterns. Theoretical saturation was considered to have been reached, as no new themes emerged during the final interviews.

## Results

The results are presented according to objectives (primary and secondary) and variables of interest, rather than by methodological approach. In this way, qualitative and quantitative approaches are combined to clarify and assess the selected evaluation criteria.

### Descriptive statistics

#### Participants

Inclusion took place between December 2022 and April 2023, with the study concluding in July 2023. Due to the group-based nature of the intervention, all participants had to start simultaneously. Although 24 were initially planned, only 15 could be recruited and consented within the limited post-treatment inclusion window (3 weeks to 3 months). This reflects recruitment constraints rather than participant dropouts.

Individual clinical characteristics of each participant are presented in Table 3.

**Table 3:** Individual clinical characteristics.

Participant	Age at diagnosis	Type of surgery^a^	Axillary procedure^b^	Treatment^c^^,^^d^
P1	37	T	SN	R; H
P2	56	T	SN	R; H
P3	42	IMR	SN	H
P4	52	M	AD	C; R; H
P5	36	M	AD	NC; R; H
P6	42	T	SN	C; R; H
P7	58	M	0	H
P8	48	M	AD	C; R; H
P9	61	T	SN	R; H
P10	49	T	SN	C; R; H
P11	48	T	SN	R; H
P12	54	T	SN	NC; R; H
P13	58	T	SN	R; H
P14	47	T	SN	R; H
P15	53	T	SN	R; H
	Mean 50 (±7.48), [ min 37- max 62]	Left breast 60%/Right breast 40%		

a M, mastectomy; IMR, immediate reconstruction mastectomy; T, tumorectomy; 2 participants received post-operative rehabilitation. (13.3%).

b AD, axillary dissection; SN, sentinel node; 0, None.

c C, adjuvant chemotherapy; NC, neoadjuvant chemotherapy; R, radiotherapy; H, hormone therapy.

d Excluding hormone therapy.

Two participants withdrew their consent during the program due to health problems and family difficulties.

#### Feasibility

Feasibility was supported by the percentage of sessions completed. Participation in Aqua Polo sessions was analyzed in terms of individual rates of completed sessions. The median participation rate during the study period was 88%, with values ranging from 25% to 100%. Among participants, 86.7% (13/15) achieved a participation rate of 60% or higher, corresponding to all participants who completed the intervention; the two participants below this threshold were those who withdrew for family-related reasons. The 90% bilateral confidence interval ranging from 64% to 100% (*p* = 0.0003 for the test H0: *p* ≤ 40%).

##### From reasons for engagement to overall program satisfaction

The initial intention of participation was to engage in meaningful actions, such as “*overcoming the disease”* (P3W1) and even “*getting out of it”* (P10W1), getting back into action and “*starting a new life”* (P8W1). The prescribing physicians’ recommendations on the post-cancer benefits of PA provided a compelling argument for participation: “*I agreed to take part in the Aqua Polo program because Dr. X informed me about the benefits of water sports in reducing the risk of disease recurrence*” (P1W1). Participants aimed to adopt healthier behaviours, discover a new activity, to step out of their comfort zone with the determination to finish the programme: “*I'm going to discover myself because I've never gone beyond my limits, [..] to push myself a bit, because it's something I would never have done. So yes, for me, it's a big personal challenge”*. (P1W1).

Overall, participants appreciated the type of sporting activity, its intensity and difficulty, the gradual nature of the sessions and the resulting sense of well-being. The design of the experience, including planning, organization, and patient monitoring, was also valued, with participants feeling “*well supported* [Editor's note: by the supervisory team]” (P2W1). One noted, “*I was also surrounded by health professionals who adapted their actions according to the pathology”*. (P3W1).

A strong initial motivation was the search for social connections, particularly meeting others who had been through similar experiences. Support from those around them was generally positive and encouraging. Over time, participants felt increasingly effective, and the group dynamic proved to be attractive and continually growing, contributing to their satisfaction. The gradual formation of a collective also drove commitment: “*When you're in a team, you rely on each other, you say to yourself ‘gosh, they're relying on me and I'm not going this week’. It's not even an effort, I said to myself: ‘You're at least trying in relation to the people who have made a commitment to you'”* (P8W8). Participants were intrinsically motivated by competition and challenge, which encouraged them to surpass themselves, improve technically, and gain energy for everyday life. They also enjoyed the pleasure of practicing a sport “*as before”*, intending to complete the experience: “*I'm back to* [the] *same person I was before: sporty, dynamic, happy. Last but not least,* [I have rediscovered] *the desire to carry on.”* (P10W1).

Participants agreed on the importance of formalizing the scientific legacy of the experiment to generalize and share it widely. They also recognised the interest and appeal of water polo, a new sporting activity, and encouraged its broader introduction: “*We're pioneers, so if it's good for us, it will be good for others, and that's what healing is all about, and I think we will all feel good, so it will be good for others”.* (P13W8).

##### Initial emotions, projections and representations

The sport was primarily perceived as one with collective values: “*exchange, sharing the game to try to progress together because it's easier in a group.”* (P4W1). Its aquatic nature made it attractive due to the benefits for the body. The sport was seen as demanding and exclusive: “*If it were a sport, very easy and accessible, it would perhaps be less useful and less interesting for us”* (P4W8). It was practiced in a place renowned for its sporting excellence, with Olympic swimmers and coaches, and local fame.

These factors generated mixed emotions: pride and enthusiasm about participating, but also fear of not being up to the task, being judged, or exposing their bodies: “*There*’*s the challenge of* [Author’s note: wearing a swimming costume and exposing the body] *but I'm going to do my best to feel good without putting on four layers of* [make-up]*”* (P1W1).

##### Obstacles and suggestions for improving the experience

Participants identified several practical, physical, and psychological barriers that could affect attendance and engagement in the programme. Practical constraints included the compatibility of training sessions with family and professional responsibilities, as well as the time and travel required to reach the venue. As one participant explained, “It was mainly challenging for my professional life, because it was quite difficult to fit into my schedule” (P2W1). Geographical accessibility was also an issue: “It is still right in the city centre, and for me it takes 45 min each way” (P8W1). Participants also expressed concerns related to the physical and psychological consequences of treatment and to the aquatic environment. These included fear of exacerbating existing pain: “[I am afraid of experiencing] pain that is too severe” (P10W1) and discomfort associated with prolonged exposure to chlorinated water: “Because of the medically induced menopause, my skin is dry, and I am concerned about spending two hours in chlorinated water” (P6W1).

To improve the experience, participants suggested providing introductory meetings and informal social activities to facilitate group cohesion from the outset. They also recommended a more balanced distribution of training phases to preserve sufficient energy for technical exercises. Finally, some felt that complementary physical activity could support their progression and expressed a need for greater encouragement to engage in such activities alongside the Aqua Polo sessions.

### Physical and psychosocial outcomes

Given the feasibility-oriented nature of this study and the limited sample size, the statistical analyses were not designed to formally assess effectiveness, but rather to explore exploratory findings that may inform future investigations. To complement this exploratory approach, the qualitative findings were modelled to highlight the main emerging categories and their dynamics, as illustrated in Figure 2.

**Figure 2:**
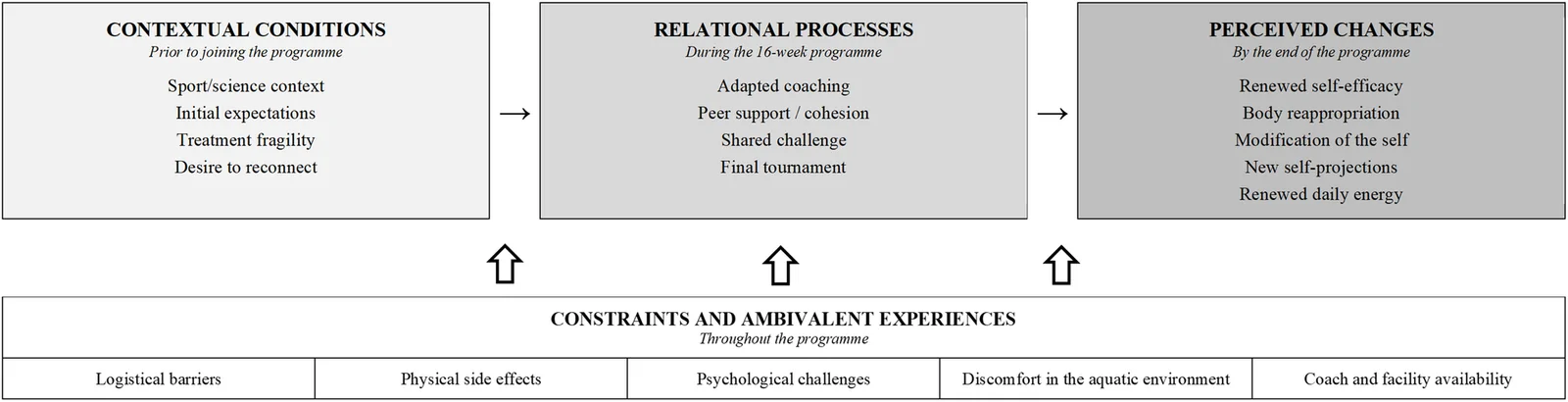
Thematic diagram of qualitative categories: contextual conditions, relational processes, and perceived outcomes of the aqua polo programme.

#### Physical capacities and fatigue

The mixed model analysis indicated decreases in heart rate during the Step-Test and one minute after test but not after three minutes. There was also a trend towards a decrease in fat mass and a significant increase in lean mass, with overall weight remaining stable throughout the study. Exploratory analyses of the Revised Piper Fatigue Scale did not reveal significant changes over the intervention period. Quadriceps strength measurements were excluded from data processing, as the test used proved to be imprecise, leading to flawed and inconsistent data collection (Table 4).

**Table 4:** Physical capacities summaries and mixed models.

Characteristic	Baseline^a^ (*n* = 15)	W4^a^ (*n* = 11)	W8^a^ (*n* = 13)	W10^a^ (*n* = 13)	W12^a^ (*n* = 13)	W16/end of program^a^ (*n* = 13)	Random slope [95% CI] (*p*-value for significance)
Heart rate at rest (bpm)	91 [54–122]			87 [50–120]		87 [60–110]	−0.25 [−0.67,0.17] (*p* = 0.22)
Heart rate post exercise (bpm)	127 [88–162]			110 [83–150]		116 [87–148]	−0.78 [−1.51,−0.05] (*p* = 0.04)
Heart rate 1 min post exercise (bpm)	104 [69–135]			87 [58–120]		92 [64–112]	−0.85 [−1.45,−0.26] (*p* = 0.009)
Heart rate 3 min post exercise (bpm)	84 [52–129]			78 [56–111]		85 [62–105]	−0.35 [−1.17,0.46] (*p* = 0.37)
Fat mass (kg)	29.9 [11.7–41.2]	29.2 [14–39.3]	26.8 [13.5–40]		22 [13.8–36.3]	20 [8.4–37.5]	−0.43 [−0.91,0.04] (*p* = 0.07)
Lean mass (kg)	42.2 [33.6–50.6]	41.5 [25.3–51.4]	42 [32–50.6]		42.7 [33.3–54.4]	42.5 [37.5–57.5]	0.13 [0.03,0.23] (*p* = 0.01)
Weight (kg)	61.7 [46.5–89.5]	62.6 [46.5–89.2]	60.9 [46.1–70.1]		60 [46.2–88.9]	59.2 [46.6–89.6]	−0.15 [−0.78,0.48] (*p* = 0.60)
Amplitude of arm movement^b^ (external rotation) (degree)	170 [110–180]			180 [110–180]		180 [110–180]	−0.03 [−0.67,0.62] (*p* = 0.93)
Amplitude of arm movement^b^ (abduction) (degree)	70 [10–180]			70 [30–90]		75 [40–90]	−0.5 [−2.14,1.21] (*p* = 0.56)

a Displayed statistics are medians [mix-max].

b Treated breast side.

#### Psychosocial outcomes

##### Towards a modification of the self

The programme was experienced as a suspended time, “*something for* [oneself]*”* (P15W16), offering an escape from daily life and a sense of calm. It fostered pride, fulfilment and self-esteem: “*People look at me differently.* [I see] *admiration in their eyes.”* (P6W8). This supported psychological and social recovery, marked by “*strength, courage and hope”* (P1W16), and a more positive self-image: “*meeting other people who have unfortunately been through it, but who, if they take part in the study, are doing well, so that also gives hope”* (P1W1).

Recovery *from* the disease became tangible through new skills and personal breakthroughs. “*For me, who has been followed by psychiatrists, psychologists and whatever else you want for X months, this, in 3 months, has been my greatest therapy”.* (P1W16). Sharing with peers helped put difficulties into perspective, spark solutions, and refocus on the essentials: “*It's all about well-being: all the friends we've made, being able to talk about our illness and everything else.* [Sport gave] *my body a workout, it helped me get out of the dark fog I was in”* (P11W8).

##### The role of competition

The final tournament, a unique feature of the programme, sparked strong and ambivalent emotions – apprehension, excitement, pride, and relief: “*It was crazy; I felt like I was going to take a school exam. I was nervous, thinking, ‘Am I going to play well? Be perfect? What will [my family] and everyone watching think of me?’”* (P11W16).

Despite the stress, showcasing their progress in front of others was seen as a meaningful milestone in their recovery: “*We said it with the girls: ‘ win or lose, whether we score or not, we've already won everything’.”* P4W16. Each participant gave personal meaning to the event, highlighting its symbolic and collective value: “*It's the climax, and also the end in a way, but we all share together. For me, it was a game”* (P8W16).

##### The quality of life

The mixed model analysis of the QOL questionnaire (QLQ-BR23) indicated higher scores over time in the “sexual quality of life” dimension, rated as BRSEE (BReast SExual Enjoyment). The median score was 66.7 [33.3–100] at baseline, remained stable at 66.7 [33.3–100] at week 10, and increased to 100 [33.3–110] by the end of the program. The random slope for BRSEE was 1.48, with a 95% confidence interval of [0.08, 2.88], indicating statistical significance (*p* = 0.04).

Exploratory analyses indicated higher arm symptom scores (BRAS) over time, with median [range] values increasing from 11.1 [0–77.8] at baseline to 27.8 [0–66.7] at week 10 and remaining at 22.2 [0–100] at the end of the programme (random slope = 1.54, 95% CI 0.38–2.72; *p* = 0.01). Analyses of the other QOL endpoints did not reveal any significant changes over time. However, qualitative findings also suggested broader quality-of-life benefits, notably through renewed body perception and improved energy to manage chronic fatigue.

##### Reclaiming your body

Illness and treatment often affected body image: “*There's the menopause [caused by] the treatment, the weight gain: it's not easy to cope when you put on 4/5 kilos”* (P2W1). Wearing a swimsuit could trigger discomfort and self-comparison: “*I have one breast that's smaller than the other and somehow I don't accept my breast, I ignore it. So putting myself in a swimming costume..* *I'm making progress, but I struggle with my image, my body. For me, yes, it's a challenge”.* (P10W1).

Participants described the programme as helping them manage treatment-related weight gain and work towards a more satisfying appearance. Gradually, self-image began to heal: “*I accept my body more, not that I feel beautiful, but since I've been doing this I've been optimizing my physique”* (P9W8).

The group offered a safe space, helping participants overcome inhibitions and rebuild self-confidence: “*I was very angry with my body a year ago. Because it gave me a fatal disease, so I rejected it. Now I'm reclaiming and using it. We're showing off in our swimmingsuits [..] I tell myself I can do the exercises like the others. I'm not a failure, I am worth something. So overall my self-esteem is better”* (P1W8).

##### An energizing activity in a context of chronic fatigue

Exploratory quantitative analyses did not indicate significant changes in fatigue or sleep. However, participants consistently described post-exercise tiredness as “good fatigue”, unlike that caused by hormone therapy. They emphasized the benefits of aquatic activity – improved tone, mobility, and physical capacities – and the satisfaction of breaking out of a sedentary lifestyle. This sense of renewed energy was often described in contrast to their pre-programme state: “*It really got me out of there. I feel like I've come back to life, and that it's given me a new lease of life. If I hadn't done this programme, I wouldn't have evolved. I wouldn't have moved”* (P10W3).

##### New self-projections

The illness shifted participants’ mindset: they now prioritize themselves, take on new challenges, adopt healthier habits, and begin to distance themselves from cancer. “*It's the will to live. The rage to live and to have overcome the disease*” (P1W16). They reported that the programme contributed to this shift – boosting self-confidence, creating momentum, and encouraging continued PA to sustain the benefits.

For some, the collective experience also generated new, positive memories: “*We were unlucky enough to have cancer, and lucky enough to take part in the project”* (P15W16). Overall, it marked a turning point: the end of treatment and the beginning of a gradual return to normality.

##### A renewed sense of competence and efficacy

Over time, participants described a growing sense of self-efficacy, supported by a professional sports environment and a pedagogical approach with adapted goals. The coaches were praised for their rigour and guidance, enabling progress in a demanding sport like water polo, regardless of age or treatment after-effects: “*It's good to do a sport not everyone can do. It proves you that anything is possible, that you can surpass yourself, always go further [..] It restores confidence in your body. I know how to do it. And you're proud of what you've achieved”* (P6W8).

Individual and collective progress in the water was associated with a renewed perspective on their experience: “*the cancer didn't stop everything”* (P2W8). This renewed sense of competence extended beyond the pool, helping some better manage fatigue and re-establish daily routines.

## Discussion

### Feasibility and acceptability of the programme

The study showed that an adapted water polo program delivered 3 weeks to 3 months after completion of chemotherapy and/or radiotherapy was feasible, with 87% of participants attending at least 60% of the sessions. Notably, all participants who completed the programme achieved the predefined adherence threshold of ≥60% of sessions. These findings align with the *acceptability* and *demand* dimensions of feasibility described by Bowen et al. (14) as participants consistently expressed high levels of satisfaction and strong interest in the programme. The study was not powered to assess efficacy because of the small sample size and absence of a control group. However, qualitative findings highlighted the importance of social support, sought by 93% of participants, and its role in motivating regular physical activity. This is consistent with previous research showing that perceived support and physician recommendations influence adherence to physical activity (26, 27).

Exploratory quantitative findings suggested favourable trends in aerobic capacity and body composition. While these results were not derived from confirmatory statistical testing, they are consistent with existing evidence on the benefits of post-cancer physical activity (28, 29) and provide preliminary observations to inform future controlled trials. Participants frequently highlighted the credibility of the sports venue and the professionalism of the multidisciplinary team, which facilitated initial implementation. In a research perspective oriented toward implementation in similar community-based sports clubs, these findings offer valuable insights for the future scaling and adaptation of the intervention.

The diversity of motivational drivers identified – ranging from health and enjoyment to challenge and social commitment – also reflects the importance of tailoring such programmes to a variety of intrinsic and extrinsic motivations (30, 31). As highlighted in sports psychology literature, creating a social environment focused on athlete well-being is essential (32). In the present study, this was supported by assembling a multidisciplinary team that blended top-level sports coaching with the expertise of adapted PA teachers and paramedical professionals, including physiotherapists and sports psychologists. Research on the Dragon Boat experience (33), emphasizes the value of environments centered on mastery objectives and friendly competition. Appropriate challenges, personalized mentoring, positive feedback, and collective celebration may encourage participation. This reinforces the value of hybrid sports activities that draw on familiar disciplines (e.g., football, handball) while adapting to the specific needs of diminished populations (e.g., walking football, colpbol). As McDonough et al. (34) note, such activities shift focus away from the illness, fostering relationships around a shared and positive objective (sport). This provides a strong foundation for effective support during remission. Future research should assess the multidimensional benefits of these adapted practices across diverse pathologies and populations.

Participants were included during the early recovery period following chemotherapy and/or radiotherapy, when treatment-related effects may persist. These include chronic fatigue, sleep disorders, muscle and joint pain, functional limitations, and cardiorespiratory impairment (35). Participants reported a decline in QoL, along with cognitive and emotional consequences. In this context, PA, particularly adapted sports, has been shown to mitigate these side effects (36). It is also possible that the early timing of inclusion, during a particularly vulnerable recovery phase, partly explains the limited improvements observed in CRF and QoL scores. However, participants qualitatively described the physical benefits of the program, such as improved heart rate during exercise (validated by the step test), toning, and overall physical well-being. Water polo, among the most demanding aquatic sports, engages the entire body through dynamic, coordinated movements requiring strength, aerobic and anaerobic endurance, and cardiovascular fitness (37). These preliminary findings suggest that water polo, when progressively introduced and tailored to participants’ physical capacities, may represent an interesting sporting option for women recovering from breast cancer.

### Adapted sports activity as a lever for psychological growth

In addition to physical benefits, PA significantly enhances psychological well-being and daily life quality (38, 39). Qualitative findings suggest that participants may begin to envision the post-cancer period more positively, gradually shifting their focus away from the illness. While the framework of post-traumatic growth (PTG) was not used to structure the coding process, several emergent themes aligned with PTG dimensions, which were identified inductively through participants’ interviews. PTG encompasses five key areas: greater appreciation of life, improved relationships, personal strength, new life possibilities, and spiritual development (40). PA promotes PTG by influencing these dimensions through mechanisms like self-perception, social experiences, philosophical reappraisal, and health outcomes (13, 41). Our exploratory findings are consistent with those of Chen et al. (41), suggesting that the programme may support processes consistent with PTG. Sabiston et al. (42) highlighted the importance of social support among Dragon Boat participants for regaining control, developing an ‘athlete identity’, and overcoming physical challenges. Similar elements emerged in participants’ experiences of adapted water polo.

Aqua polo, as a “team sport”, may encourage socialization through shared experiences, helping participants bond around a demanding activity. Research by Carter et al. (43) confirms that team-based PA improves cohesion and program adherence. Discourse analysis suggests that cooperation and shared commitment may enhance collective effectiveness, reinforcing personal mastery and well-being. This relational social support may contribute to participants’ social development and sustained PTG (44). In this regard, the final tournament both reinforced group cohesion and generated emotional stress. Future programs should address this challenge through coaching strategies that frame it as a collective goal, supporting team identity and turning stress into shared progress.

### Study limitations

The Aqua polo programme faced several challenges that impacted recruitment and implementation. Fixed session dates required rapid, simultaneous inclusion, but the narrow eligibility window (3 weeks to 3 months post-treatment) restricted access to eligible participants. As a result, the target of 24 was not reached, and some could not complete all 16 sessions. In addition, the feasibility design, small sample size, and absence of a control group preclude conclusions regarding the effectiveness of the intervention. These recruitment and design constraints should be considered when planning future multicentre trials.

Implementation also relied on external sports structures, which introduced several logistical constraints. These included coach availability linked to competition schedules and limited access to facilities due to public opening hours and club time slots. These factors should be considered when adapting or scaling up similar programmes, as they may limit the transferability of the findings to other populations and healthcare settings.

The qualitative findings should also be interpreted in light of possible expectation and social desirability biases. As participants voluntarily enrolled in this novel community-based sport programme, they may have been particularly motivated or positively predisposed towards the intervention. Nevertheless, the analysis also retained contrasting experiences, including pain, fatigue, logistical constraints, concerns about body exposure, and stress related to the final tournament.

Measurement tools for quadriceps strength revealed inconsistencies, with significant fluctuations tied to the assessor and force produced, raising concerns about their reliability.

The increase in BR23 arm symptom scores warrants particular attention. This subscale captures upper-limb pain, swelling, and difficulty in arm movement, and therefore reflects a broader symptom burden than shoulder mobility alone. No intervention-related adverse events were reported through the study safety monitoring procedures. Nevertheless, the increase in patient-reported arm symptoms highlights the need for systematic monitoring of upper-limb symptoms in future studies. Given the absence of a control group, these findings cannot determine whether symptom changes were related to the intervention, pre-existing treatment-related morbidity, or their natural evolution during recovery.

Two participants withdrew for family-related reasons unrelated to the intervention. As all absences and withdrawals were systematically documented, the risk of uncontrolled missing data was minimized, supporting transparency in the analysis.

### Clinical and research implications

Beyond feasibility indicators, this feasibility study provides exploratory insights into the motivational and relational dynamics supporting engagement. Participants described peer support as important for their psychological well-being. They also highlighted the coach's role in creating a safe and motivating environment that supported their confidence and sense of legitimacy as athletes and survivors. Diverse motivational drivers (health, curiosity, social connection, and challenge) emerged as important levers for adherence and behaviour change. Although not powered to demonstrate efficacy, trends in aerobic capacity and body composition suggest potential physical benefits. Broader inclusion criteria could facilitate recruitment in future implementations. Home-based or hybrid training options may also help integrate PA into daily life, support personalisation, and prevent injury. Integrating a physiotherapist and offering tailored home exercises based on patient history may help reduce musculoskeletal discomfort. These elements provide concrete directions for scaling and adapting such programmes in real-world, community-based contexts.

## Conclusion

This study supports the feasibility and acceptability of implementing an adapted water polo programme post-breast cancer, although the small sample size precludes conclusions regarding effectiveness, exploratory quantitative findings and qualitative experiences suggest potential physical and psychosocial benefits. The qualitative findings also provided valuable insights into peer support, coaching relationships, and motivational processes that may facilitate engagement with physical activity. Overall, these findings reinforce the value of community-based, multidisciplinary approaches and support further action-research to refine and scale such interventions in real-world contexts.

## Data Availability

The raw data supporting the conclusions of this article will be made available by the authors, without undue reservation.
